# Supplementary material for: Single-cell absolute contact probability detection reveals chromosomes are organized by multiple low-frequency yet specific interactions
Source: Nat Commun. 2017 Nov 24;8:1753. doi: 10.1038/s41467-017-01962-x (PMC5700980; doi:10.1038/s41467-017-01962-x)
Supplement: Supplementary file 1 — Supplementary Information [file 41467_2017_1962_MOESM1_ESM.pdf]

Table of Contents

Supplementary Notes
- Oligopaint probesets
  - Supplementary Table 1: Oligopaint probe sets for libraries.
- PCR primers and secondary oligonucleotides
  - Supplementary Table 2: Unlabelled PCR primers
  - Supplementary Table 3: Labelled PCR primers
  - Supplementary Table 4: Secondary labelled oligos
- Probes synthesis
- ModEncode data files used in this study
  - Supplementary Table 5: ChIP-chip/seq data files employed in this study
- Correlation of replicates of late-embryo and S2 Hi-C interaction maps
  - Supplementary Table 6: Correlation of Hi-C replicates
- Supplementary Figure 1
- Supplementary Figure 2
- Supplementary Figure 3
- Supplementary Figure 4
- Supplementary Figure 5
- Supplementary Figure 6

References

Supplementary Notes

Oligopaint probesets

Supplementary Table 1: Oligopaint probe sets for libraries.

Chr	Lib	Genomic coordinates		Coverage (kb)	Number of oligos	Specific primer pairs	
2L	1	5265000	5285000	20	304	BB287-FWD	BB288-REV
2L	2	5320000	5340000	20	310	BB293-FWD	BB294-REV
2L	3	5520000	5550000	30	530	BB295-FWD	BB296-REV
2L	4	5715000	5745000	30	475	BB84-FWD	BB83-REV
2L	5	9990000	10010000	20	267	BB287-FWD	BB288-REV
2L	6	10180000	10210000	30	405	BB293-FWD	BB294-REV
2L	7	10420000	10540000	120	1615	BB295-FWD	BB296-REV
2L	8	10710000	10750000	40	516	BB84-FWD	BB83-REV
2L	9	10980000	11010000	30	488	BB193-FWD	BB280-REV
2L	10	11100000	11130000	30	523	BB82-FWD	BB278-REV
2L	11	11265000	11295000	30	522	BB81-FWD	BB281-REV
2L	12	11500000	11530000	30	480	BB298-FWD	BB187-REV
2L	13	12995000	13025000	30	30	AB_12-FWD	AB_13-REV
3R	14	12260000	12330000	70	944	BB291-FWD	BB292-REV
3R	15	12450000	12480000	30	405	BB300-FWD	BB301-REV
3R	16	12840000	12960000	120	1541	BB302-FWD	BB303-REV
2L	IT-17	10065000	10095000	30	482	BB291-FWD	BB292-REV
2L	IT-18	10265000	10295000	30	479	BB300-FWD	BB301-REV
2L	IT-19	10600000	10630000	30	462	BB302-FWD	BB303-REV

PCR primers and secondary oligonucleotides

Fluorophore-labelled PCR primers, 5’-phosphorylated PCR primers used in the lambda exonuclease protocol and DNA secondary oligos purified by using high-performance liquid chromatography were purchased from IDT (Coralville, US) . Unlabelled, unphosphorylated primers purified by using standard desalting, were also purchased from IDT.
Supplementary Tables 2-4 display the list of PCR primer pairs and secondary oligos used in this work.

Supplementary Table 2: Unlabelled PCR primers

Name	Sequence	Lib	Chr
BB287-FWD	/5Phos/CGCTCGGTCTCCGTTCGTCTC	1	2L
Sec1-BB288-REV	CACCGACGTCGCATAGAACGGAAGAGCGTGTGGGGCTAGGTACAGGGTTCAGC	1	2L
BB293-FWD	/5Phos/CCGAGTCTAGCGTCTCCTCTG	2	2L
Sec1-BB294-REV	CACCGACGTCGCATAGAACGGAAGAGCGTGTGAACAGAGCCAGCCTCTACCTG	2	2L
Sec5-BB294-REV	TAGCGCAGGAGGTCCACGACGTGCAAGGGTGTAACAGAGCCAGCCTCTACCTG	2	2L
BB295-FWD	/5Phos/GCGTTAGGGTGCTTACGTCTG	3	2L
Sec1-BB296-REV	CACCGACGTCGCATAGAACGGAAGAGCGTGTGCACCTCCGTCTCTCACCTCTC	3	2L
Sec5-BB296-REV	TAGCGCAGGAGGTCCACGACGTGCAAGGGTGTCACCTCCGTCTCTCACCTCTC	3	2L
BB84-FWD	/5Phos/GATACGTTGGGAGGCAATGAG	4	2L
Sec1-BB83-REV	CACCGACGTCGCATAGAACGGAAGAGCGTGTGATCCTAACAATCCCGCTGAGG	4	2L
Sec5-BB83-REV	TAGCGCAGGAGGTCCACGACGTGCAAGGGTGTATCCTAACAATCCCGCTGAGG	4	2L
BB287-FWD	/5Phos/CGCTCGGTCTCCGTTCGTCTC	5	2L
Sec1-BB288-REV	CACCGACGTCGCATAGAACGGAAGAGCGTGTGGGGCTAGGTACAGGGTTCAGC	5	2L
BB293-FWD	/5Phos/CCGAGTCTAGCGTCTCCTCTG	6	2L
Sec1-BB294-REV	CACCGACGTCGCATAGAACGGAAGAGCGTGTGAACAGAGCCAGCCTCTACCTG	6	2L
Sec5-BB294-REV	TAGCGCAGGAGGTCCACGACGTGCAAGGGTGTAACAGAGCCAGCCTCTACCTG	6	2L
BB295-FWD	/5Phos/GCGTTAGGGTGCTTACGTCTG	7	2L
Sec1-BB296-REV	CACCGACGTCGCATAGAACGGAAGAGCGTGTGCACCTCCGTCTCTCACCTCTC	7	2L
Sec5-BB296-REV	TAGCGCAGGAGGTCCACGACGTGCAAGGGTGTCACCTCCGTCTCTCACCTCTC	7	2L
BB84-FWD	/5Phos/GATACGTTGGGAGGCAATGAG	8	2L
Sec1-BB83-REV	CACCGACGTCGCATAGAACGGAAGAGCGTGTGATCCTAACAATCCCGCTGAGG	8	2L
Sec5-BB83-REV	TAGCGCAGGAGGTCCACGACGTGCAAGGGTGTATCCTAACAATCCCGCTGAGG	8	2L
BB193-FWD	/5Phos/TTGATCTCGCTGGATCGTTCT	9	2L
Sec5-BB280-REV	TAGCGCAGGAGGTCCACGACGTGCAAGGGTGTGGGAGTAGGGTCCTTTGTGTG	9	2L
BB82-FWD	/5Phos/GTATCGTGCAAGGGTGAATGC	10	2L
Sec1-BB278-REV	CACCGACGTCGCATAGAACGGAAGAGCGTGTGGAGCAGTCACAGTCCAGAAGG	10	2L
BB81-FWD	/5Phos/ATCCTAGCCCATACGGCAATG	11	2L
Sec5-BB281-REV	TAGCGCAGGAGGTCCACGACGTGCAAGGGTGTGGACATGGGTCAGGTAGGTTG	11	2L
BB298-FWD	/5Phos/CGTCAGTACAGGGTGTGATGC	12	2L

Sec1-BB187-REV	CACCGACGTCGCATAGAACGGAAGAGCGTGTGTTGATCTTGACCCATCGAAGC	12	2L
AB_12-FWD	/5Phos/TCGGCCCTTATCGGTAGCAG	13	2L
Sec1-AB_13-REV	CACCGACGTCGCATAGAACGGAAGAGCGTGTGCAACGCGCTCGTGTACAACG	13	2L
BB291-FWD	/5Phos/CAGGTCGAGCCCTGTAGTACG	14	3R
Sec1-BB292-REV	CACCGACGTCGCATAGAACGGAAGAGCGTGTGCTAGGAGACAGCCTCGGACAC	14	3R
BB300-FWD	/5Phos/CCAGTGCTCGTGTGAGAAGTC	15	3R
Sec1-BB301-REV	CACCGACGTCGCATAGAACGGAAGAGCGTGTGCTGCAGAGAAGAGGCAGGTTC	15	3R
Sec5-BB301-REV	TAGCGCAGGAGGTCCACGACGTGCAAGGGTGTCTGCAGAGAAGAGGCAGGTTC	15	3R
BB302-FWD	/5Phos/CGCACTGAACCAGACTACCTG	16	3R
Sec1-BB303-REV	CACCGACGTCGCATAGAACGGAAGAGCGTGTGGAGAGGCGAGGACACCTACAG	16	3R
Sec5-BB303-REV	TAGCGCAGGAGGTCCACGACGTGCAAGGGTGTGAGAGGCGAGGACACCTACAG	16	3R
BB291-FWD	/5Phos/CAGGTCGAGCCCTGTAGTACG	17	2L
Sec1-BB292-REV	CACCGACGTCGCATAGAACGGAAGAGCGTGTGCTAGGAGACAGCCTCGGACAC	17	2L
BB300-FWD	/5Phos/CCAGTGCTCGTGTGAGAAGTC	18	2L
Sec1-BB301-REV	CACCGACGTCGCATAGAACGGAAGAGCGTGTGCTGCAGAGAAGAGGCAGGTTC	18	2L
Sec5-BB301-REV	TAGCGCAGGAGGTCCACGACGTGCAAGGGTGTCTGCAGAGAAGAGGCAGGTTC	18	2L
BB302-FWD	/5Phos/CGCACTGAACCAGACTACCTG	19	2L
Sec1-BB303-REV	CACCGACGTCGCATAGAACGGAAGAGCGTGTGGAGAGGCGAGGACACCTACAG	19	2L
Sec5-BB303-REV	TAGCGCAGGAGGTCCACGACGTGCAAGGGTGTGAGAGGCGAGGACACCTACAG	19	2L

Supplementary Table 3: Labelled PCR primers

Name	Sequence
BB506-A647 (Sec1)	/5Alex647N/CACCGACGTCGCATAGAACGG
BB506-A488 (Sec1)	/5Alex488N/CACCGACGTCGCATAGAACGG
BB510-Cy3B (Sec5)	/5Cy3B/TAGCGCAGGAGGTCCACGAC

Supplementary Table 4: Secondary labelled oligos

Name	Sequence
Sec1-A647-X2(Sec1)	/5Alex647N/CACACGCTCTTCCGTTCTATGCGACGTCGGTGagatgttt/3AlexF647N/
Sec1-A488-X2(Sec1)	/5Alex488N/CACACGCTCTTCCGTTCTATGCGACGTCGGTGagatgttt/3AlexF488N/
Sec5-Cy3B-X2(Sec5)	/5Cy3B/ACACCCTTGCACGTCGTGGACCTCCTGCGCTAagatgttt/3Cy3B/

Probes synthesis

Multiplexed libraries were amplified using emulsion PCR with universal primers following the steps and protocols described elsewhere [1] (http://genetics.med.havard.edu/oligopaints). Oligopaints probes containing secondary oligo binding sites were synthesized using the lambda exonuclease method [2].

ModEncode data files used in this study

A description of the genome-wide ChIP-chip/seq data files used for assignment of chromatin state is provided in Supplementary Table 5.

Supplementary Table 5: ChIP-chip/seq data files employed in this study

Cell type	Epigenetic mark	File ID
**Late Embryo**	*H3K4me3*	modENCODE_5096
	H3K27me3	modENCODE_3955
	PC	modENCODE_3957
**Early Embryo**	*H3K4me3*	modENCODE_789
	H3K27me3	modENCODE_3811
	PC	modENCODE_5064
**S2**	*H3K4me3*	modENCODE_914
	H3K27me3	modENCODE_298
	PC	modENCODE_326

Correlation of replicates of late-embryo and S2 Hi-C interaction maps

Supplementary Table 6: Correlation of Hi-C replicates

Experiment	Read	Reads	Mapped	%	Mapped int.	%	Valid int.	%	Dangling	%	Extra DE	%
Late embryo replicate 1	1	771,584,699	543,089,230	70.4								
	2	771,584,699	523,164,711	67.8	461,642,603	59.8	113,867,043	14.8	189,398,549	41	50,386,592	11
Late embryo replicate 2	1	765,716,879	503,352,249	65.7								
	2	765,716,879	479,743,113	62.7	416,797,286	54.4	168,158,279	22.0	131,890,044	32	18,994,806	5
S2 replicate 1	1	619,637,214	449,119,241	72.5								
	2	619,637,214	437,937,471	70.7	382,574,953	61.7	92,244,799	14.9	166,503,128	44	24,627,012	6
S2 replicate 2	1	768,189,050	473,607,172	61.7								
	2	768,189,050	464,792,365	60.5	415,009,752	54.0	38,522,335	5.0	231,820,094	56	91,873,204	22
S2 replicate 3	1	440,441,503	287,855,072	65.4								
	2	440,441,503	281,915,992	64.0	225,500,438	51.2	79,568,795	18.1	69,733,515	31	7,648,210	3

Experiment	Read	Duplicates	%	R. breaks	%	Over rep.	%	Error	%	Sel circle	%	Final merge valid
Late embryo replicate 1	1											
	2	222,013,421	48	122,332,379	26	6,873,614	1	132,48	0	1,068,660	0	
Late embryo replicate 2	1											
	2	109,469,472	26	112,554,684	67	6,499,838	2	128,89	0	413,190	0	**282,025,322**
S2 replicate 1	1											
	2	158,379,607	41	137,160,088	36	9,403,744	2	560,41	0	1,570,343	0	
S2 replicate 2	1											
	2	271,699,414	65	114,735,347	298	8,462,059	2	377,26	0	632,010	0	
S2 replicate 3	1											
	2	68,269,233	3	70,259,409	88	4,916,355	2	401,56	0	202,378	0	**210,335,929**

Supplementary Figure 1

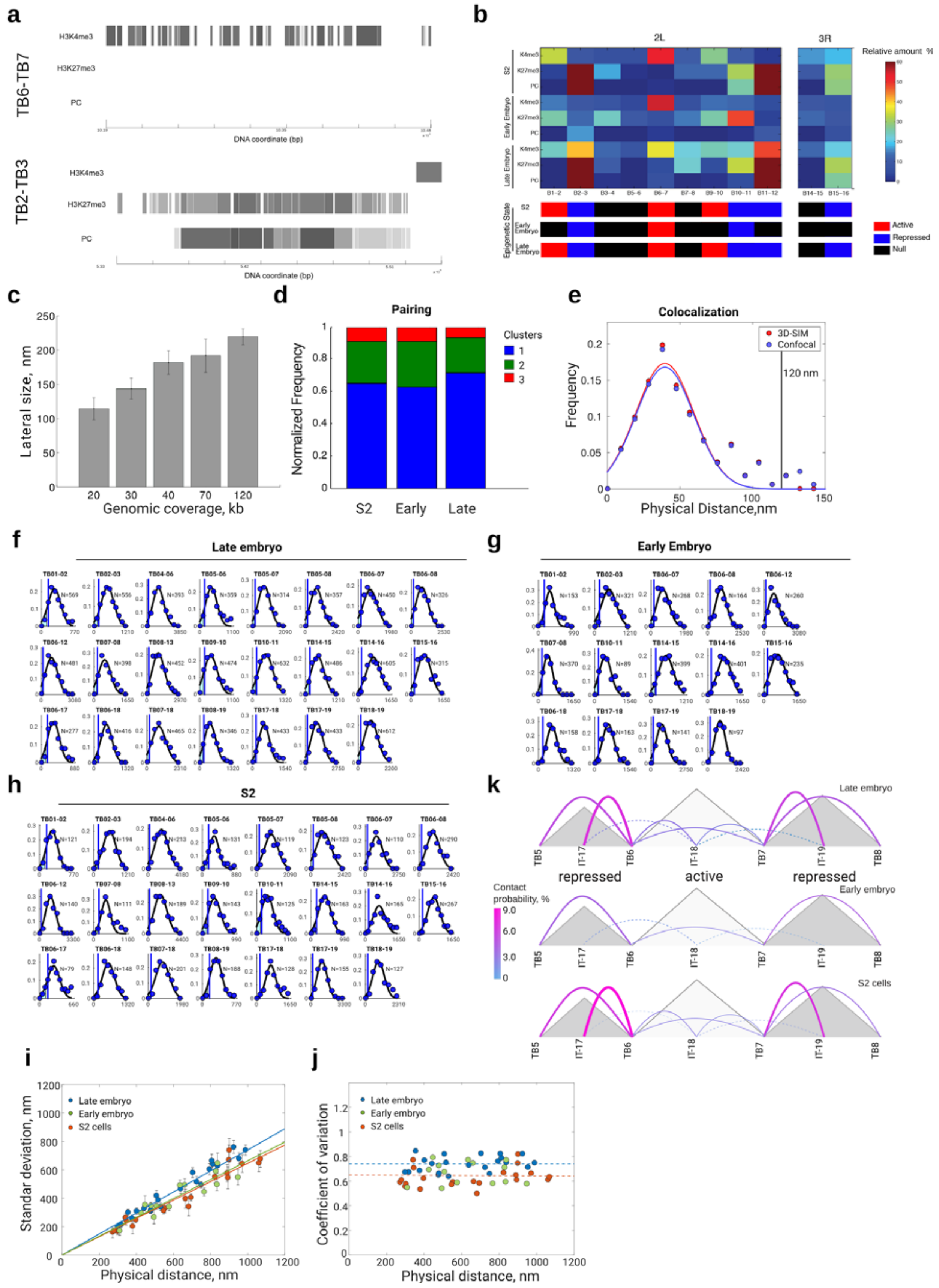

(a-b) Classification of TADs chromatin states. **(a)** shows representative examples of active (top) and repressed (bottom) chromatin states. Chip-Seq profiles for H3K4me3, H3K27me3 and Polycomb for TADs between borders TB6-TB7 and TB2-TB3 (upper and lower panels) are depicted. Abscissas indicate the genomic coordinates between the barriers. Grey intensities in Chip-seq profiles are proportional to intensity of the detected peak according to the color-coded scale on the right. **(b)** Upper panel shows the relative amount for each mark between barriers for each cell type calculated as defined in the paragraph below. Colorcode in scale bar on the right is proportional to the relative amount of H3K4me3, H3K27me3 and PC. Lower panel depicts the resulting chromatin state of each TAD depending on the proportions of each epigenetic mark (see paragraph below 'Definition of chromatin states') defining active (red), inactive (black) and repressed (blue) chromatin states. ChIP-chip/seq computed peaks were downloaded from ModEncode (ftp://data.modencode.org/D.melanogaster/) and datasets used are described in Supplementary Table 5.

Definition of chromatin states

Epigenetic states of TADs encompassed between consecutive oligoPAINT libraries were classified into three categories: active, inactive and repressed, based on enrichment of histone modifications and Polycomb from ChIP-chip/seq profiles for each cell type (early embryonic, late embryonic and S2 cells) obtained from the modENCODE database [3]. Active chromatin TADs were selected based on the relative amount of H3K4me3. Repressed TADs were selected based on the relative amount of H3K27me3 and Polycomb (PC) proteins. Inactive TADs were selected based on the total or partial depletion of H3K4me3, H3K27me3 and PC proteins. In each case the relative amount for each mark was calculated as the ratio between the number of base pairs bound by each epigenetic mark over the total number of base pairs between consecutive libraries. Domains containing more than 25% of a given mark and less than 25% of the remaining marks were considered as enriched for that particular mark (*e.g.* region between TB1 and TB2 for S2 cells displays H3K4me3 = 34%, H3K27me3= 3%, PC = 0, then the region is defined as enriched for H3K4me3 and defined as active TAD). Regions having less than 25% enrichment for any mark were considered as inactive. To confirm our results, enrichment for marks in each region was also visually inspected using a homemade Matlab routine and Gbrowse (GBrowse.org).

(c) Population average lateral size of foci as a function of the genomic size of the 19 oligopaint libraries employed in this work. Error bar represents the standard deviation of the sizes distribution obtained from a Gaussian fitting.

(d) Homologous pairing for the different cell types. We estimated the pairing of chromosomes by counting the total number of foci detected for each Oligopaint library per single cell from 3D-SIM imaging. Pairing was similar for all cells types and above 60%. For simplicity the population average for all libraries is displayed. Y coordinates indicate the relative frequency of detection of foci while the x coordinates indicates the type of cell line analyzed. A total of 5130, 1310 and 1882 cells were computed for the analysis in late embryo, early embryo and S2 cells, respectively. We note that early and late embryonic cells are diploid, while S2 cells present typically four copies of each chromosome [4].

(e) Distribution of distances for TAD border TB2 labelled in two colors and imaged either by confocal microscopy or by 3D-SIM. Both distributions are identical with median and standard deviation of 42 and 26 nm, respectively. The black solid line at 120 nm includes >99% of total observations. N = 161 and N = 166 for 3D-SIM and confocal microscopy measurements, respectively. From these experiments, we calculate the precision of localization in our measurements at 40 nm and define that any two TAD borders located at less than 120 nm are co-localizing.

Note on the colocalization precision in 3D-SIM and the selected threshold to calculate absolute contact probability.

Colocalization between multicolor fluorescent beads yields the maximal precision of colocalization that can be measured, in our conditions, by 3D-SIM for isotropic objects of sizes smaller than the resolution limit and located at the surface of the coverslip when imaged in two distinct channels (colocalization = 30 ± 5 nm, mean ± SD). This value, that reveals the minimal distance at which two objects can be considered as colocalizing, is rather ideal and arises from bright objects located at the surface of the coverslip. Thus, it does not take into account the effect of low signal and background noise and depth-dependent spherical aberrations introduced for objects further away from the objective. To determine the precision of colocalization between libraries and to emulate an equivalent situation to that employed for all libraries imaged in this work, we fluorescently labelled the same library with two spectrally-different fluorophores and measured their separating distance. In our experimental conditions the minimal distance that indicates that two libraries colocalize is (42 ± 26 nm, mean ± SD, i.e. a co-localization precision 30% lower than in ideal conditions). In a conservative approach, we decided to employ the

results obtained from a single library labelled with two colors and the upper limit of the distribution obtained to measure the absolute probability of contact between borders. Then from the Gaussian fitting we assume that two libraries have 99% chances of co-localizing if their separating distances is less than 120 nm (i.e. three standard deviations away from the mean).

Increasing the distance threshold to calculate contact probability does not increase significantly the measured frequency of interaction.

Contacts detected through chromosome conformation capture techniques such as Hi-C can be located in the 3D space at distances ranging from tens to a few hundreds of nanometers [5]. To ensure that the low probability of contacts between borders obtained with our method was not due to a selection of threshold that could underestimate the mapped distances by Hi-C, we increased systematically the value of the threshold starting at 100 nm in steps of 50 nm. For all the values tested the probabilities increased as expected but remained low reaching (18.2±2.6%, mean±SEM) in average for the largest threshold of 300 nm.

(f-h) Distribution of distances for all pairs of libraries computed in this work for late (**f**) and early (**g**) embryos, and for S2 cells (**h**). In all panels, the Y axis indicates the relative frequency while the X axis indicates the physical distances measured. A Gaussian fit (black line) was used to determine the mean and standard deviation. Blue vertical solid lines represent the colocalization threshold (120 nm) for a single library labelled with two colors with a 99% confidence interval as described in Fig. S1e. Light blue shaded area indicates the integral under the curve for each gaussian fitting to calculate the absolute contact probability. The number of each pair of libraries computed is indicated on top of each panel and N represents the number of cells analyzed from at least three biological replicates.

(i) To get further insight into the mechanism of chromatin folding within epigenetic domains, the standard deviation of distance measurements between barriers was plotted as a function of the mean physical distance between borders for all three cell types. Notably, the relation between these quantities was linear and independent of chromatin type for all cell types. Note that the slope was slightly higher for late embryo than for early embryo and S2 cells (0.74 ± 0.03, 0.66 ± 0.05, 0.65 ± 0.03 nm/nm, respectively), indicating that for equivalent mean size of TADs their structure displays higher variability in late embryonic cells. Error bars represent the standard error of the mean (SEM) as obtained from bootstrapping, by randomly resampling

with replacement each dataset one hundred times to estimate the errors in standard deviations

(j) Coefficient of variation for physical distances between borders as a function of the mean physical distance for all three cell types. Dotted lines represent the average for late embryo (0.74, light blue) and S2 cells (0.65, orange).

(k) Schematic representation of contact probability between and within TADs (solid colored lines) as depicted in the main figure for late embryo and S2 cells and including early embryo (middle panel). Size of triangles representing TADs (grey shaded) is proportional to genomic length (scale bar on top). Chromatin type is indicated at the bottom of each TAD. Thickness of the lines is proportional to the absolute contact probability with values depicted in color coded scale bar on the right. Dotted lines indicate inter-TADs contact probabilities.

Supplementary Figure 2

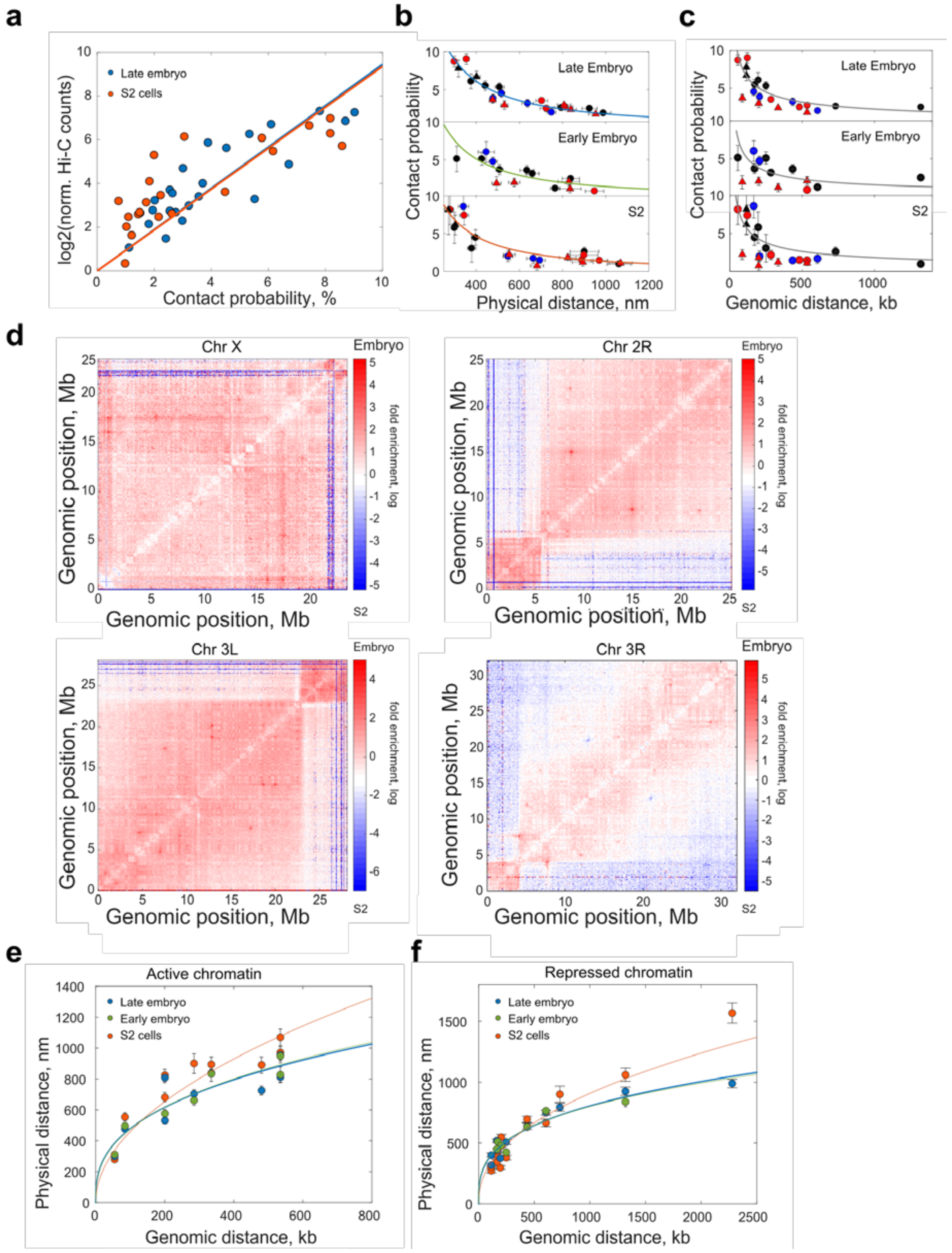

(a) Log_2 normalized Hi-C counts *vs.* microscopy absolute contact probability for consecutive and non-consecutive TAD borders for embryo (light blue) and S2 cells (orange). Blue and orange line represent a linear fitting for late embryo and S2 cells. Note that both cell types display equivalent non-linear relations between Hi-C and microscopy measurements.

(b) Absolute contact probabilities as a function of mean physical distance for consecutive and non-consecutive TAD borders for late embryo, early embryo and S2 cells. Chromatin state of domains encompassed by probes is color-coded as follows, red: active, blue: repressed, black: inactive. Error bars in the x-axis represent the SEM on the physical distances distribution whereas error bars in the y-axis represent the SEM as obtained from bootstrapping, by randomly resampling with replacement each dataset one hundred times to estimate the error in contact probabilities.

(c) Circles are employed when the pair of libraries is at borders and triangles are employed when at least one of the libraries is within a TAD. Experimental data was best described by a power-law $P = \alpha\, d_{3D}^{-\theta}$ (solid lines reproducing color code of Fig. 2b of the main text). The pre-factor α was obtained from the best fitting parameters values of late embryo (α = $1.6E^4$) and kept constant for the other fittings. The scaling exponent best fitting values were: $\boldsymbol{\theta}$ = 1.31 ± 0.01, $\boldsymbol{\theta}$ = 1.35 ± 0.03, $\boldsymbol{\theta}$ = 1.36 ± 0.02 for late embryo, early embryo and S2 cells, respectively.

(d) Absolute contact probabilities as a function of genomic distance with chromatin state colors and symbols as in Supplementary Fig. 2b. Solid lines are a guide-to-the-eye. Note that given the different degree of compaction between chromatin states (see Fig. 2e-f and Supplementary Fig. 2d), for equivalent genomic distances the contact probability for active chromatin is systematically lower than that of inactive/repressed chromatin domains.

(e) Matrix of relative frequency of normalized Hi-C counts for late embryo *vs.* S2 cells for chromosomes X, 2R, 3L, and 3R. Scale Bar represents the logarithmic ratio of the contact frequencies between cell types. Resolution=50 kb.

(e-f) Plots of mean physical distance between pairs of oligopaints libraries flanking active **(e)** or inactive/repressed chromatin **(f)** as a function of genomic distance. The lines indicate a power-law fitting ($d_{3D} = \gamma d_{kb}^{\beta}$), with the exponent β as displayed in Fig 2e-f. The pre-exponential factors were γ=88 ± 25, γ=93 ±35 and γ=41 ± 25 for late embryo, early embryo and S2 cells, respectively. Circles are employed when the pair of libraries are at barriers while triangles are employed when at least one of the libraries is within a TAD. Error bars represent ± SEM.

Supplementary Figure 3

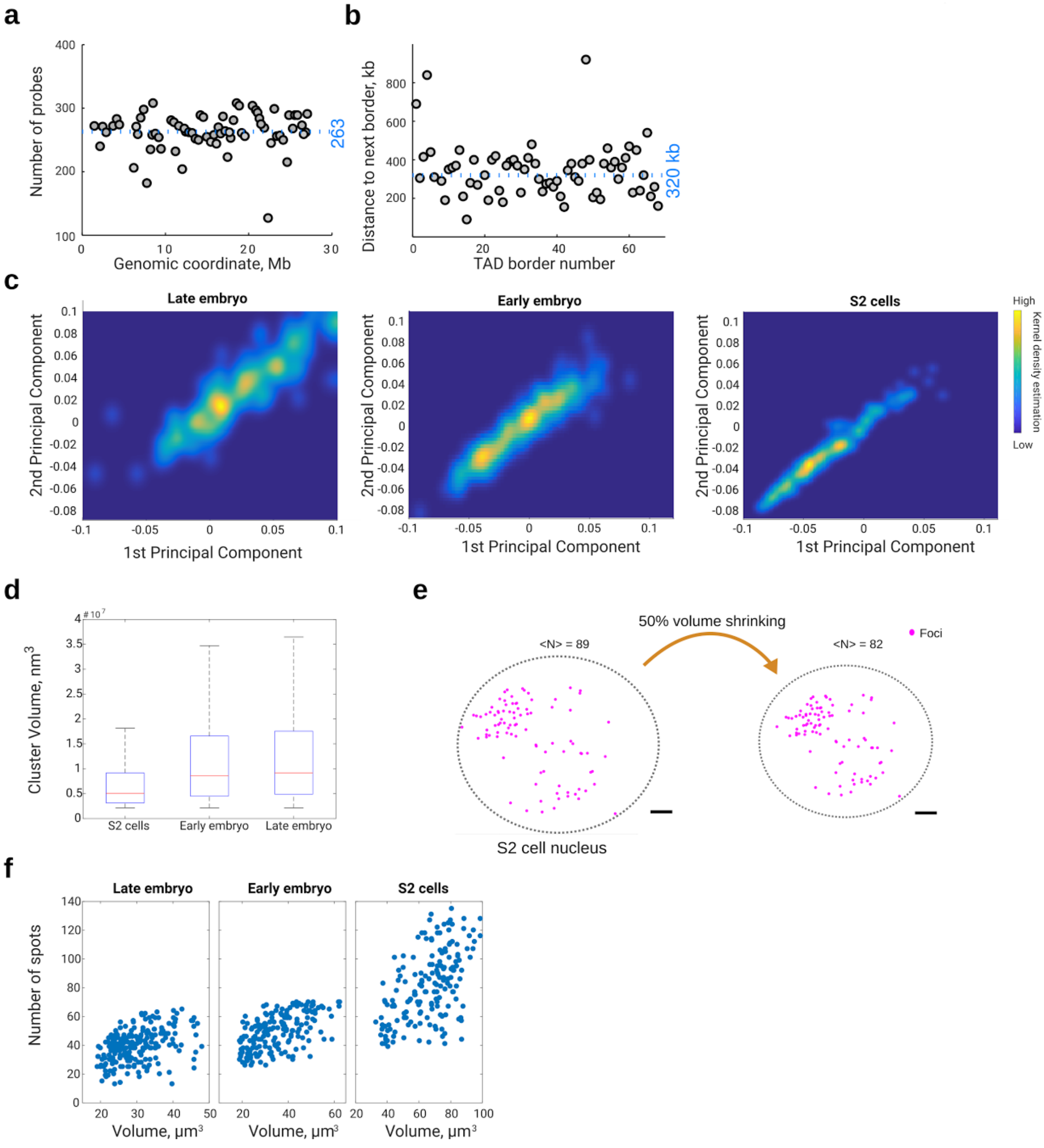

(a) Total number of probes used for each library as a function of the genomic coordinate. The average number of probes is 263.

(b) Genomic distance between consecutive TAD borders as a function of border number. Barriers are numbered sequentially from centromere to telomere. The distribution of distances between barriers is homogeneous, with a mean of 320 kb.

(c) Principal Component Analysis (PCA) of the normalized p(r) distributions from Fig. 3b, displaying the first two principal components scores of every cell. Color-code indicates the kernel density estimation heatmap, the density is calculated on the

number of points in a location, with larger number of clustered points resulting in larger values. Blue: low, yellow: high.

(d) Average size of foci detected from simultaneously labelling 69 barriers in chromosome 3R. Note that the volume per foci increases from S2 cells to early and late embryo respectively, consistent with data shown in Fig. 3.

(e) The lower number of detected foci in embryonic cells is not associated with their reduced nucleus volume with respect to S2 cells. Left, clusters detected after segmentation of a representative S2 cell. Right, same cell after volume shrinkage of 50%. <N> indicates the average number of clusters detected before and after nucleus volume reduction for a representative cell. Scalebar=1 μm. The difference in detected number of cluster cannot account for the differences observed between cell types. To test more quantitatively if the reduced number of clusters observed in embryonic cells was due to the reduced nucleus volume of embrionyc cells (50% reduction with respect to S2 cells) combined with the inability of 3D-SIM to resolve foci separated by distances below the resolution limit (~120 nm in xy, ~250 nm in z), we computationally reduced the total volume occupied by all foci in S2 cells. To this end, the radial distance of all detected foci respect to the center of mass was reduced by 20% (equivalent to a 50% reduction in volume in a perfect sphere) and next we quantified the number of foci that remained at a distance resolvable by 3D-SIM. The number of foci detected after volume shrinking diminished by less than ~7%. The latter confirms that the lower number of detected foci in early and late embryos arises from a higher frequency of interaction between TAD borders and not from the smaller volume of cells or limitations in the resolutive power of 3D-SIM.

(f) Number of detected spots as a function of nuclear volume for each cell. Mean volume is (32 ± 13) μm^3, (35 ± 17) and (67 ± 23) μm^3 for late embryo, early embryo and S2 cells, respectively. N=180.

Detailed R_g and D_{max} values from Figure 3b of the main text

	Rg	Dmax
**Late embryo**	1.6 ± 0.3	4.0 ± 0.9
**Early embryo**	1.9 ± 0. 3	4.9 ± 0.1
**S2 cells**	2.6 ± 0.4	6. 4 ± 0.9

Discussion on the total number of foci imaged when labelling 69 TAD borders in 3D-SIM

The total number of barriers appearing as foci in microscopy imaging can be estimated as $N = 69 \times \sum_{k=1}^{3} p_k \, x \, k)$, where p_k represents the relative frequency of cells displaying k foci. From the pairing frequency obtained from single border labelling (Supplementary Fig. 1c) and assuming the absence of long-range interactions between TADs borders, the predicted maximum number of foci for each cell type is 92, 100 and 97 for late embryo, early embryo and S2 cells, respectively.

Supplementary Figure 4

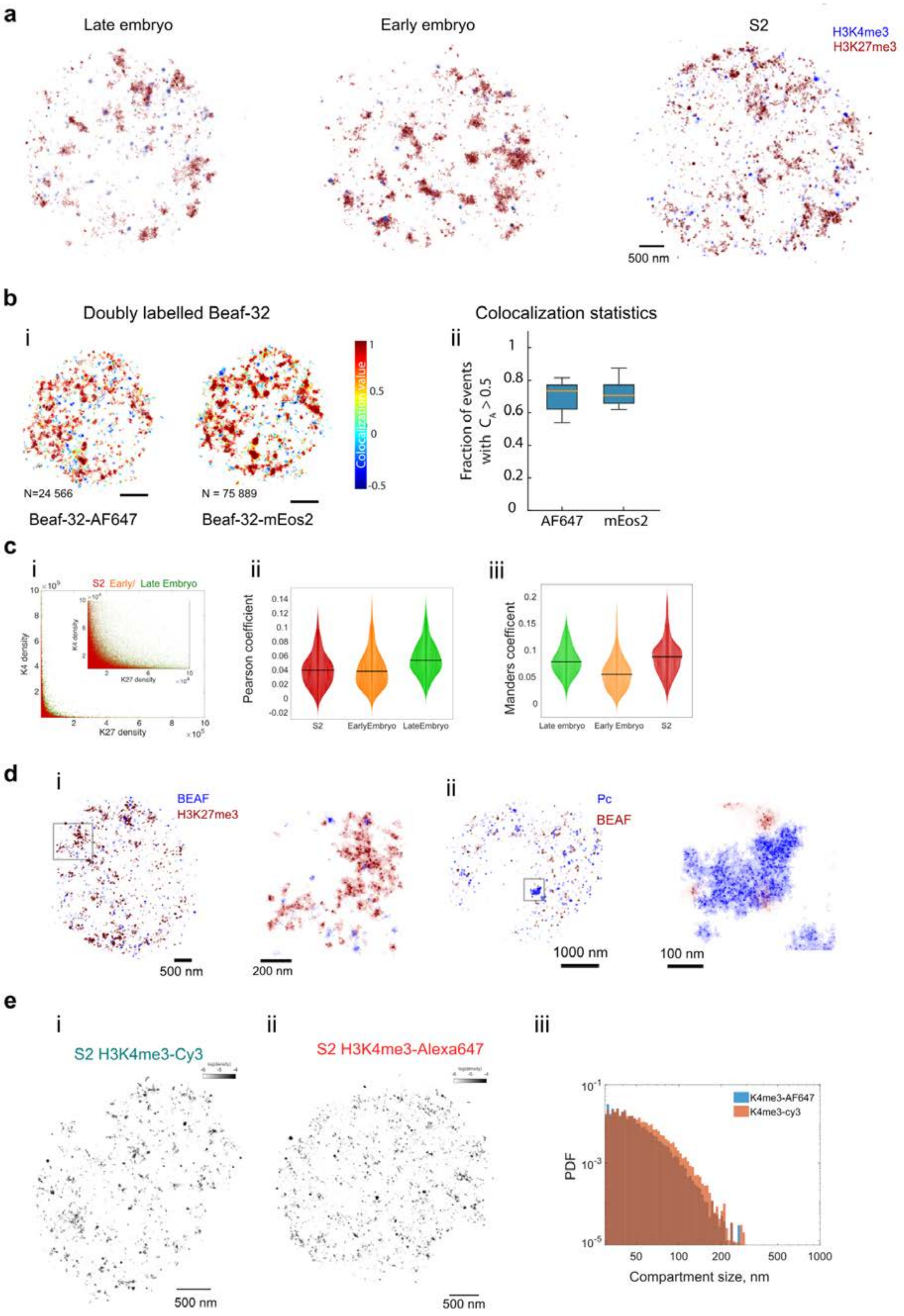

(a) Two-color dSTORM image of active (H3K4me3, red) and repressive (H3K27me3, blue) chromatin marks in representative late embryo, early embryo and S2 cells. Scalebar=500 nm.

(b) (i) Two-color SMLM imaging of Beaf-32. Beaf-32 was labeled directly using a fusion protein (Beaf-32-mEos2) and by immunofluorescence (primary Beaf-32 antibody conjugated to AF647). Cells were then imaged sequentially in these two channels and analysed using the aCBC analysis [6]. Left panel shows the aCBC map of the AF647 channel, and right panel displays the aCBC map of the mEos2 channel. The numbers (N) at the bottom of each cell correspond to the number of single detections in each map obtained from 20,000 frames and are an indication of the density of events. The color scale on the right (Colocalization values) reflects the aCBC coefficient for each single localization. Values above 0.5 indicate a high degree of colocalization. N=14. Scale bars: 1 µm (ii) Statistics of the colocalization for the Beaf-32-AF647 and Beaf-32-mEos2 channels. Boxplots indicate the median (orange bar), 25th and 75th percentile limits (blue areas), and extreme values (whiskers) of the fraction of events with aCBC colocalization value (C_A) > 0.5.

(c) (i) Pixel-by-pixel colocalization analysis between active and repressed histone marks. The data distribution into two separate groups, showing varying signal levels of one mark with little or no signal from the other, indicates a very low level of colocalization between single molecule events. (ii-iii) Quantification of co-occurrence between active and repressive chromatin compartments using the Pearson's and Mander's colocalization coefficients criteria for S2, early and late embryonic cells. . Note that for all type of analysis active and repressed marks display very low overlap for all cell types.

(d) (i) Representative two-color dSTORM image of Beaf-32 (blue) and H3K27me3 (red) in a S2 cell. Inset on the right is a magnification of the region selected by the gray square. Beaf-32 rarely colocalizes with H3K27me3 marks. (ii) Typical two-color dSTORM image of Polycomb (blue) and Beaf-32 (red). Beaf-32 does not colocalize with Polycomb. Quantification of the degree of co-localization of Beaf-32 vs. H3K27me3 and Polycomb using aCBC analysis, yielded values of 0.23 ± 0.04 and 0.18 ± 0.04 respectively, confirming the absence of colocalization between this marks.

(e) (i-ii) Representative images of single-color dSTORM imaging of H3K4me3 labelled with antibodies bearing different organic fluorophores (either Cy3b or Alexa 647, N=35). Scalebar = 500 nm. (iii) Distribution of H3K4me3 compartment sizes for either AF647 or Cy3b. From these experiments, we conclude that no significant differences

in the spatial localization, and distribution of sizes of compartments were observed when Cy3b was used instead of AF647.

Supplementary Figure 5

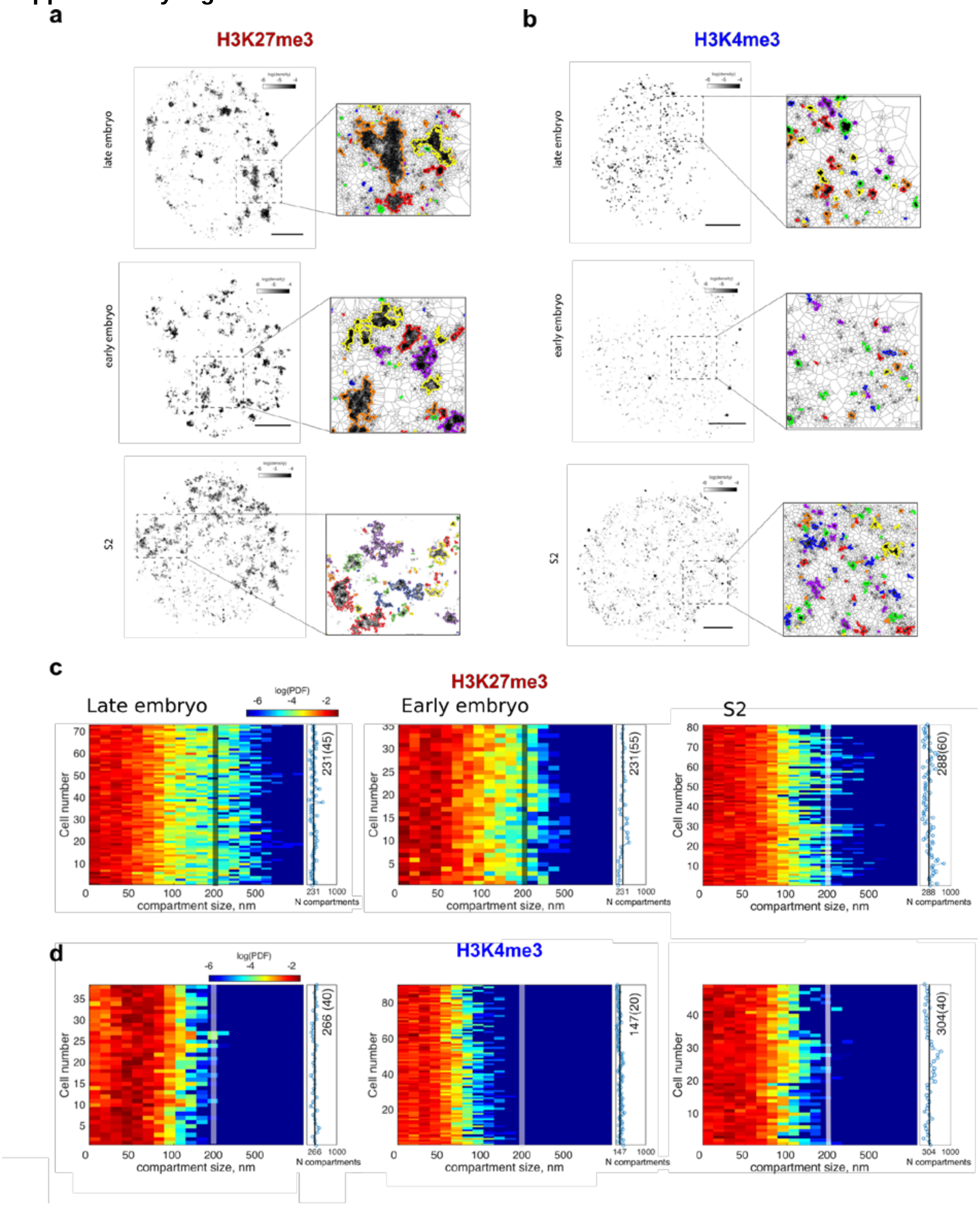

(a-b) Representative dSTORM images of Alexa-647 labelled H3K27me3 **(a)** and H3K4me3 **(b)** for all cell types employed in this work. Images show density maps computed from the area of the polygons obtained from the Voronoï diagram with color-coded scale defined at the top. Scalebar = 1 µm. Zoomed regions displaying detected domains (highlighted with different colors) using automatic segmentation on the basis first-rank density criteria as defined in [7].

(c-d) Single cell distribution of H3K27me3 **(c)** and H3K4me3 **(d)** compartment sizes, defined as equivalent diameters in nm, for late and early embryonic and S2 cells. Each horizontal line represent the distribution from a single cell. Vertical lines at 200 nm are used as a reference to compare different histograms. Note that x-scale and colormap are in logarithmic scales (see colorbar above). Right panel to each distribution shows the number of compartments detected per cell. The mean is displayed as a vertical black line and the value and its standard deviation (in parenthesis) are quoted.

Supplementary Figure 6

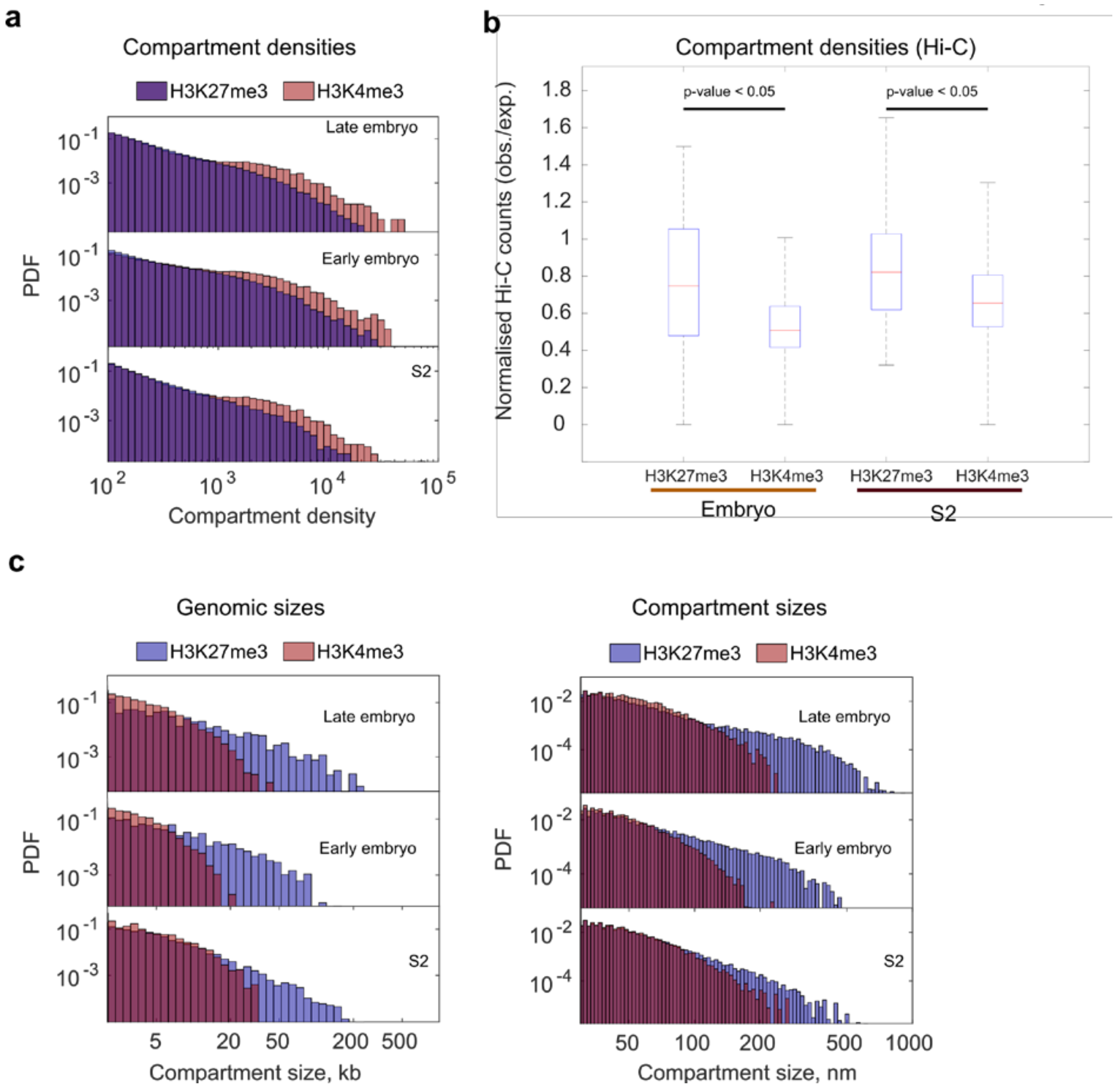

(a) Population based distribution of compartments densities for H3K4me3 and H3K27me3 chromatin marks for all cell types studied in this work. Compartment density is defined as the number of single-molecule detections divided for the area of the compartment. PDF is probability density function. Note that for all cell types, repressed mark compartments display higher densities than active compartments in good agreement with local chromatin folding measurements (see Fig. 2e-f in the main text). The density of compartments, both active and repressed, is higher in embryonic cells with respect to S2 cells, in good agreement with local chromatin folding data (Fig. 2e-f).

(b) Boxplot of the distribution of relative Hi-C normalised counts (observed/expected) within H3K27me3 or H3K4me3 domains in embryos and S2 cells. Each entry of the Hi-C normalised matrix has been divided by the genome-wide average normalised

Hi-C counts at the corresponding genomic distance to take into account for the expected diagonal decay of the Hi-C data. We found the results to be robust over various matrix resolutions (10, 20 and 50 kb), here are shown data at XXXkb resolution. Boxes contain 50% of the data (0.67σ), and the red lines inside them mark the medians. Outliers (>3.3σ away from the mean values) are shown as black dots. P-values were calculated using the Welch t-test.

(c) Comparison between the ensemble distributions of genomic ChIP-Seq data (i) and dSTORM compartment physical sizes (ii) from H3K4me3 and H3K27me3 for all three cell-types analyzed. PDF is probability density function. The size distributions of repressed and active compartments were broad and stretched over several decades independently of cell type, with repressed compartments being systematically larger than active compartments. Repressed compartments were significantly larger in late embryos than in S2 cells, with early embryos displaying an intermediate behavior (panel b, right). In contrast, genomic distributions of repressive domains detected by chromatin immunoprecipitation methods [8] were similar for late embryos and S2 and smaller for early embryos (panel b,left), suggesting cell-specific clustering of epigenetic domains depending on developmental and transcriptional state of each cell type.

Biases in the determination of compartment sizes by 2D-dSTORM.

To estimate epigenetic compartment sizes, we used 2D-dSTORM imaging. In our implementation, we obtained a depth of field of ~500 nm. Thus, our 2D super-resolved images are a 2D projection of the 3D density of epigenetic domains. As the large majority of domains detected (>95%) were smaller than the depth of field, then we would only expect to slightly underestimate the size of large domains. The main conclusion from these measurements is that epigenetic size distributions are not large enough to account for the large epigenetic compartments observed by STORM. Thus, the biological conclusion is still sound despite a possible underestimation in the degree of clustering of epigenetic domains.

References

1. Beliveau, B. J., Apostolopoulos, N. & Wu, C.-T. Visualizing genomes with Oligopaint FISH probes. *Curr. Protoc. Mol. Biol.* **105,** Unit 14.23. (2014).
2. Beliveau, B. J. *et al.* Single-molecule super-resolution imaging of chromosomes and in situ haplotype visualization using Oligopaint FISH probes. *Nat. Commun.* **6,** 1–13 (2015).
3. Celniker, S. E. *et al.* Unlocking the secrets of the genome. *Nature* **459,** 927–930 (2009).
4. Zhang, Y. *et al.* Expression in aneuploid Drosophila S2 cells. *PLoS Biol.* **8,** e1000320 (2010).
5. Giorgetti, L. & Heard, E. Closing the loop: 3C versus DNA FISH. *Genome Biol.* **17,** 215 (2016).
6. Georgieva, M. *et al.* Nanometer resolved single-molecule colocalization of nuclear factors by two-color super resolution microscopy imaging. *Methods* doi:10.1016/j.ymeth.2016.03.029
7. Levet, F. *et al.* SR-Tesseler: a method to segment and quantify localization-based super-resolution microscopy data. *Nat. Methods* **12,** 1065–1071 (2015).
8. Kharchenko, P. V. *et al.* Comprehensive analysis of the chromatin landscape in Drosophila melanogaster. *Nature* **471,** 480–485 (2011).
